# Postoperative Hypoalbuminemia as a Predictor of Early Mortality After Cementless Hemiarthroplasty for Hip Fractures

**DOI:** 10.3390/medicina61111936

**Published:** 2025-10-29

**Authors:** Muhammed Melez, İbrahim Altun, Ömer Can Ünlü, Kürşat Tuğrul Okur, Fırat Ozan

**Affiliations:** 1 Department of Orthopedics and Traumatology, Kayseri City Hospital, Kayseri 38080, Turkey; dr.melez@gmail.com (M.M.); omercanunlu@gmail.com (Ö.C.Ü.); firatozan9@gmail.com (F.O.); 2 Department of Orthopedics and Traumatology, Sorgun State Hospital, Yozgat 66100, Turkey; k_tugrul_okur@hotmail.com

**Keywords:** hip fractures, hypoalbuminemia, mortality

## Abstract

*Background and Objectives:* This study aimed to evaluate the factors influencing early postoperative mortality in patients undergoing cementless hemiarthroplasty for proximal femoral fractures. *Materials and Methods:* The medical records of 227 patients treated between January 2019 and December 2020 were retrospectively reviewed. Patients were divided into two groups: survivors (Group 1, *n* = 160) and non-survivors (Group 2, *n* = 67). The variables assessed included demographic data, neutrophil-to-lymphocyte ratio, surgical duration, hospital stay, American Society of Anesthesiologists (ASA) score, cardiac ejection fraction (EF), Charlson Comorbidity Index (CCI), osteoporosis status, and hemoglobin and albumin levels. Clinical evaluation was performed using the Harris Hip Score. Binary logistic regression was used to analyze risk factors; receiver operating characteristic (ROC) analysis was applied to determine cutoff values. *Results:* The mean follow-up duration was 14.03 ± 10 months. The mean ages were 80 ± 7.68 yr in Group 1 and 83.99 ± 7.42 yr in Group 2. Statistically significant differences were found between groups regarding ASA scores, intensive care unit (ICU) admission rates, and osteoporosis status (*p* < 0.001). Preoperative and postoperative albumin levels were also significantly different (*p* < 0.001). The 1-year and 6-month mortality rates were 39.6% and 29.5%, respectively. Univariate analysis identified age, EF, ASA score, preoperative and postoperative albumin levels, CCI, ICU admission, and ICU stay duration as mortality-related factors. Multivariate binary logistic regression analysis revealed that low postoperative albumin levels may have a significant effect on mortality at 1, 3, and 6 months. ROC analysis showed a significant albumin cutoff value of 2.95 g/dL. *Conclusions:* Higher postoperative albumin levels were inversely associated with early mortality following hemiarthroplasty in elderly patients. Perioperative monitoring of albumin levels may help improve outcomes, particularly in individuals with severe comorbidities.

## 1. Introduction

Most hip fractures involve the femoral neck and intertrochanteric (ITC) fractures [1]. They are preterminal injuries that can be deadly and have a substantial impact on quality of life, particularly in senior patients, due to the trauma caused by a decrease in existing bone reserve and degeneration of bone structure [2].

Despite advancements in medical and implant technology, mortality rates following hip surgery can range from 13.3% to 25% [3,4,5]. Several prognostic factors are associated with patient mortality, including hemodynamic factors, high comorbidity burden, fracture type, low body mass index (BMI), surgical factors, nutritional deficiency, low functional status or decreased mobility, advanced age, and male gender [6,7,8]. However, a relationship between inflammatory cells and mortality has been discovered in people following hip fracture surgery [9]. As a result, immune-inflammatory markers such as the neutrophil-to-lymphocyte ratio (NLR), lymphocyte-to-monocyte ratio, and C-reactive protein-to-albumin ratio have become commonly used in recent years to evaluate risk factors for mortality [10,11,12].

In elderly patients with hip fractures, hypoalbuminemia is a major risk factor for postoperative mortality and is also associated with an increased risk of postoperative infection [13,14]. Although the mechanisms underlying the association between albumin deficiency and mortality are not fully understood, several pathways have been proposed. These include its antioxidant function, its role as a nutritional marker, and its function as a negative acute-phase protein, which may amplify inflammatory responses [15]. Albumin deficiency is an independent risk factor for mortality in patients with chronic conditions, particularly chronic kidney and chronic heart disease. It is also associated with increased risks of postoperative infection, sepsis, prolonged intensive care unit stays, and intubation [16,17]. Furthermore, low albumin levels are associated with poor nutritional status and impaired healing capacity, which may exacerbate adverse outcomes [18]. Therefore, albumin, which is an indicator of an individual’s nutritional condition and overall health, may influence patient prognosis and outcome [10]. Hypoalbuminemia has been linked to increased morbidity and mortality in a wide range of medical situations, including orthopedic injuries and surgical patients [11]. Although hypoalbuminemia is widely recognized as a risk factor for postoperative mortality, limited evidence exists on the predictive value of postoperative albumin levels specifically after hip fracture surgery. Moreover, the relationship between serum albumin dynamics and time-specific mortality risk (e.g., at 1, 3, and 6 months postoperatively) has not been fully elucidated.

This study aimed to gain a better understanding of the multifactorial nature of mortality after cementless hemiarthroplasty performed in elderly patients over the age of 65 to correct hip fractures. In addition, we aimed to develop a clinical prediction model for the first 6 months using patient-related prognostic factors available at admission and after surgery to identify risk factors.

## 2. Materials and Methods

**Figure 1:**
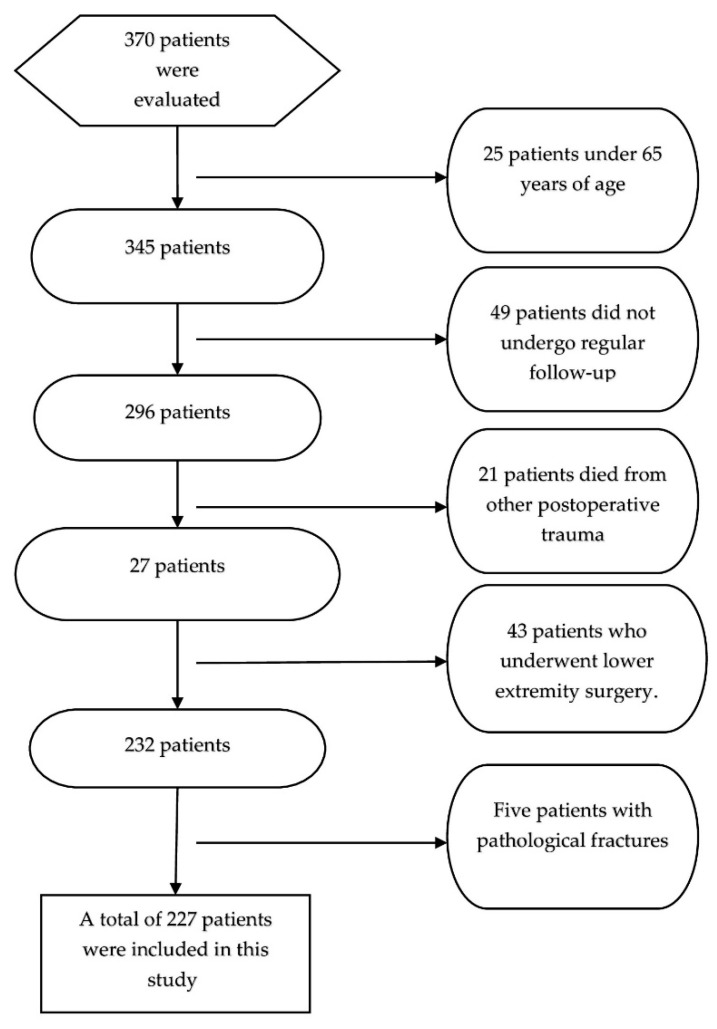
Flowchart illustrating the evaluation of patient numbers following the application of exclusion criteria.

Between 1 January 2019, and 31 December 2020, approximately 20 specialists and academic physicians performed hemiarthroplasty (TIPSAN^®^, Istanbul, Türkiye) on 370 patients with femoral proximal fractures at the Kayseri City Education and Research Hospital Orthopedics and Traumatology Clinic. Patients with acute hip fractures (presenting immediately after the fracture) caused by low-energy trauma were included as well as those who underwent cementless hemiarthroplasty after achieving hemodynamic stability, when deemed appropriate by the anesthesiologists. The study excluded patients with pathological fractures (metastatic tumor and osteomyelitis); those who had previously had surgery on the other hip, bilateral femoral proximal fractures, and periprosthetic fractures; and those who did not have regular follow-ups (Figure 1). Of the 370 patients included, 227 satisfied the study’s requirements. Patients were divided into two groups: those who survived (Group 1) and those who died during the first 6 months of follow-up (Group 2).

In addition to patients’ demographic data, the findings of the last follow-up examination and follow-up periods were obtained as well as preoperative and postoperative hemoglobin and albumin values, preoperative and postoperative NLRs, surgery duration, hospital stay, the need for postoperative intensive care, intensive care stay, the presence of additional trauma, cardiac ejection fraction (EF), and American Society of Anesthesiologists (ASA) scores. The ASA classification (scores 1–6) was used to assess patients’ perioperative risk for anesthesia. Chronic conditions were evaluated using the modified Charlson Comorbidity Index (CCI). Osteoporosis was assessed with the Singh Index and the Cortical Thickness Index, Picture Archiving and Communication Systems (PACS) (KEYDATA^®^, Information Technology Systems, Ankara, Türkiye). Serum albumin levels were measured between postoperative days 3 and 5. Low serum albumin levels were defined as <3.5 g/dL, whereas low hemoglobin levels were defined as <10 g/dL. Moreover, serum albumin levels were classified as severe (<2.5 g/dL), moderate (2.5–3.5 g/dL), or normal (>3.5 g/dL). Patients with severe albumin levels were supported with albumin infusion, and patients with moderate albumin levels were treated nutritionally. The 25-month mortality rates were calculated during the follow-up periods. Risk analyses were conducted at 1, 3, and 6 months to assess the influence of available data on mortality. Complications, reoperation demands, death dates, and mortality rates were assessed.

### 2.1. Surgery and Rehabilitation

Patients fasted for 8 h before their scheduled operation date. They received an appropriate dose of low-molecular-weight heparin every day until their operation. All patients received a 1 gr/iv prophylactic dose of first-generation cephalosporin (cefazolin sodium) half an hour before surgery. Procedures were performed with patients in the lateral decubitus position while under spinal anesthesia. In total, 6.6% (*n* = 15) of the patients underwent surgery using the Hardinge lateral technique, while 93.4% (*n* = 212) used the posterior route.

In our clinic, all patients received postoperative stockings or Jones bandages to protect them from embolism. Low-molecular-weight heparin was administered as soon as the patient was hospitalized and was issued subcutaneously at an adequate dose daily throughout the postoperative period. If there are no contraindications following discharge, the low-molecular-weight heparin treatment is continued for 35 days. Again, we recommend using embolism stockings or a Jones bandage for 4 weeks.

All patients received cefazolin sodium (1 g, intravenous) (GENSENTA^®^, Istanbul, Türkiye) as a prophylactic antibiotic. The initial preoperative dose was administered 30 min before skin incision, followed by a postoperative regimen of 1 g IV every 6 h (total of 4 g per day) throughout the hospital stay. After discharge, patients were prescribed oral antibiotics for 7 days, following institutional infection prevention protocols. After being transported to the ward following the operation, our patients were mobilized on the first postoperative day using a walker and weight-bearing on the operated side, depending on their general condition. Patients with general conditions that did not allow for movement or who required postoperative critical care were referred to the Physical Therapy and Rehabilitation Clinic on the first postoperative day for in-bed activities.

### 2.2. Statistical Analysis

After transferring to the computer system, data were analyzed using SPSS software (IBM Corp., released 2013, IBM SPSS Statistics for Windows, Version 22.0, Armonk, New York, USA). Categorical data were expressed as percentages, while continuous variables were represented by their mean values and standard deviations. Categorical variables were analyzed using the Pearson Chi-square test, and Fisher’s Exact Test was applied when the expected cell count was ≤5. The data’s conformity to the normal distribution was assessed using the Shapiro–Wilk test, Skewness and Kurtosis tests, and histogram values. The Mann–Whitey *U* test analyzed the association between continuous variables with non-normal distributions. Binary logistic regression analysis determined the factors that influenced mortality. Following the examination for multiple linearity, the data included in the model were analyzed using binary regression analysis. Gender, age, duration between hospitalization and surgery, fracture type, preoperative and postoperative NLRs, ASA, duration of surgery, CCI, EF, preoperative and postoperative albumin levels, preoperative and postoperative hemoglobulin levels, duration of hospitalization, intensive care hospitalization status, and intensive care hospitalization durations were analyzed using a single-variable binary logistic regression model. Albumin was treated as a continuous predictor. Following univariate analysis, variables such as age, ASA score, EF, CCI, postoperative albumin level, and ICU-related parameters were identified as statistically significant. These variables were subsequently entered into the multivariate logistic regression model to determine independent predictors of early mortality. As a result of this test, a risk analysis model was developed using the data that made a significant contribution, and multivariable regression analysis was performed. In the regression analysis, *p* values < 0.05 were significant. The threshold value for albumin was determined using receiver operating characteristic (ROC) analysis with SPSS. For each variable, the optimal cutoff point was determined using ROC analysis by identifying the threshold value at which the Youden Index (J), calculated as (Sensitivity + Specificity – 1), was maximized.

## 3. Results

The average follow-up time for 227 patients who underwent cementless hemiarthroplasty due to hip fracture was 14.03 ± 10 (minimum–maximum, 1–33) months. The study assessed the early effects of mortality-related factors by splitting the patients into two groups: 160 patients who survived (Group 1) and 67 patients who died within the first 6 months (Group 2). Patients in Groups 1 and 2 had mean ages of 80.17 ± 7.68 and 83.99 ± 7.42, respectively (*p* < 0.001). The study included 134 patients with femoral neck fractures and 93 patients with femoral ITC.

**Figure 2:**
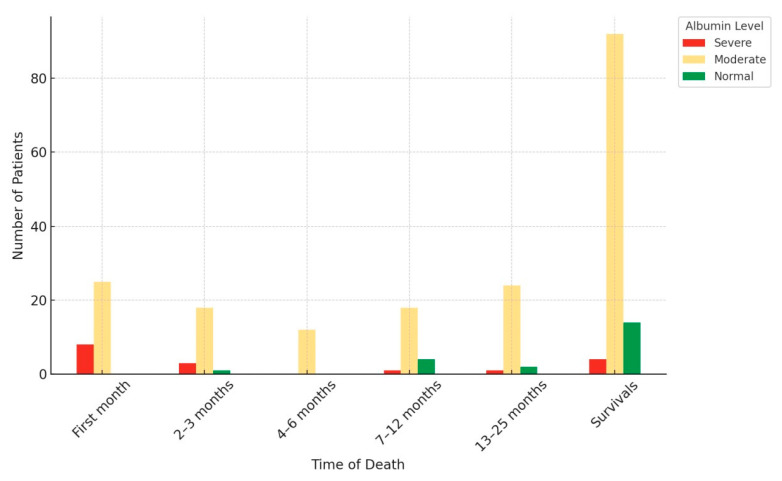
Analysis of postoperative albumin levels in surviving and non-surviving patients.

ASA scores revealed a significant difference between groups (*p* < 0.001). CCI was used to assess the impact of chronic diseases on mortality, and a substantial difference was revealed (*p* < 0.001). A total of 50.7% of patients (*n* = 115) needed postoperative intensive care hospitalization, with 43 patients (39%) in Group 1 and 72 patients (62%) in Group 2 receiving follow-up in the intensive care unit (*p* < 0.001). Osteoporosis was diagnosed in 162 patients, with a significant difference between groups (*p* < 0.001; Table 1). Within the first 6 months, 11 (64.7%) out of 17 patients had severe hypoalbuminemia, 55 (29.1%) out of 189 patients had mild hypoalbuminemia, and 1 (4.76%) out of 21 patients who presented with normal albumin levels died (Figure 2). Patients’ preoperative and postoperative albumin values (*p* < 0.001) and EF (*p* < 0.001) differed significantly between groups. In Group 2, 58% of patients had postoperative albumin levels below 29.5 g/dL compared to 27.5% of patients in Group 1 (*p* < 0.001), as shown in Table 2.

**Figure 3:**
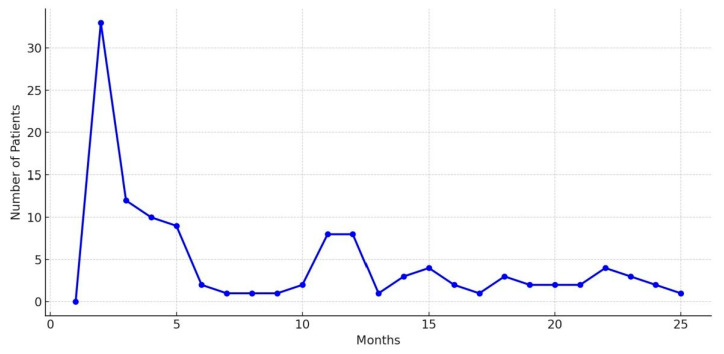
Temporal distribution of mortality among patients who non- survive the postoperative period.

The total death rate among the patients in the study was 51.5% (*n* = 117). When the survival times of the patients who died were analyzed, 14.5% (*n* = 33) died during the first month, and 5.7% (*n* = 13) died before discharge from the hospital. The mortality rate among individuals who died within the first 6 months was 29.5% (*n* = 67). In our study, the 1-yr mortality rate was determined to be 39.6% (*n* = 90; Figure 3).

**Figure 4:**
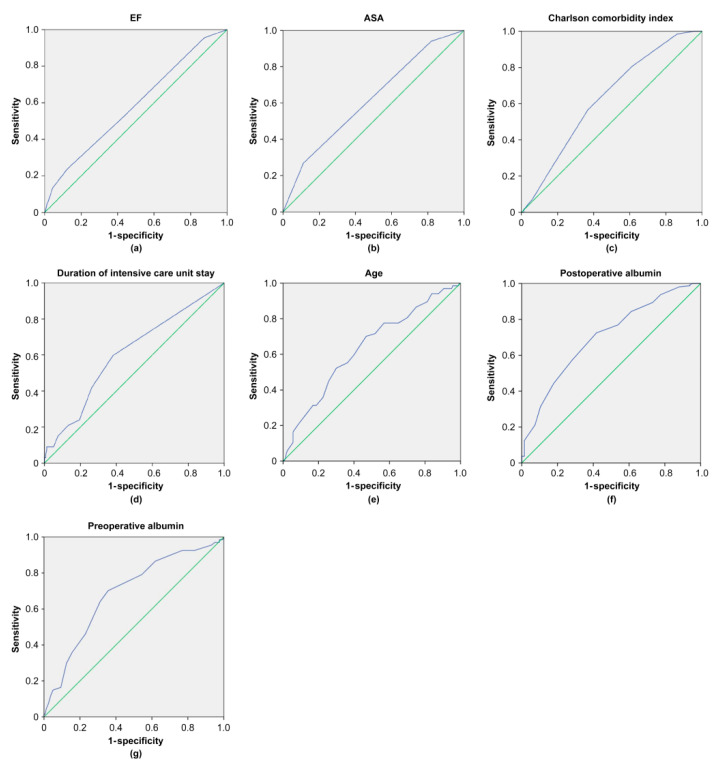
Receiver operating characteristic–based graphical representation of cutoff values for factors influencing mortality in the univariate regression analysis. (**a**) Ejection fraction; (**b**) ASA classification; (**c**) Charlson Comorbidity Index; (**d**) Duration of intensive care unit stay; (**e**) Age; (**f**) postoperative albumin; (**g**) Preoperative albumin. The blue line represents the ROC curve for each variable, and the green diagonal line represents the reference line (AUC = 0.5).

Univariate binary regression analysis was carried out for the months mentioned. Age, ASA 3, ASA 4, EF, preoperative albumin, postoperative albumin, CCI, intensive care follow-up, and length of stay in the ICU were found to be significant predictors of mortality (Table 3). Significant risk variables were identified in the first, third, and sixth months of the multivariate binary logistic regression model analysis using these parameters (Table 4). Higher postoperative albumin levels were significantly and inversely associated with the risk of mortality at 1 month (OR = 0.827; 95% CI, 0.714–0.958; p = 0.011), 3 months (OR = 0.813; 95% CI, 0.715–0.925; p = 0.002), and 6 months (OR = 0.835; 95% CI, 0.741–0.941; p = 0.003). In our risk analysis, each 1 g/dL increase in serum albumin was associated with an approximately 17.3% reduction in mortality at 1 month, 18.7% at 3 months, and 16.5% at 6 months. Although the odds ratio for one variable was high, the result did not reach statistical significance (p = 0.056), and the wide confidence interval indicated limited precision, possibly due to sample size constraints. ROC analysis was performed for covariates that significantly influenced mortality in univariate binary regression analysis (Table 5). In this analysis, the cutoff for the parameters was determined to pose a risk when age was >81.50 yr, EF was <52.5, ASA was >3, CCI was >5, ICU stay was >1.5 days, preoperative albumin levels were <3.75 g/dL, and postoperative albumin levels were <2.95 g/dL (Figure 4).

## 4. Discussion

Many risk factors have been identified for mortality after hip fracture surgery [6,7,8]. Age, gender, nutritional issues, hemodynamic variables, entrenched HAA, and other chronic conditions can increase the chance of death [6,7,8,18].

Age and gender are some of the most well-known risk variables for death [19,20,21]. Inadequate personal care for men has been linked to greater mortality rates in numerous studies [21]. According to one study, age is a relative risk for 12-month mortality [22]; however, another study found that mortality rates are high for people over the age of 80 [23]. While no significant relationship was found between gender and mortality in our study, a significant effect was identified for age and mortality, consistent with the literature, which increased by 1.08 times in patients over the age of 81.5. However, this had no significant effect on risk analysis.

CCI, ASA, and EF have direct effects on mortality rates [24]. Although CCI is effective in identifying comorbidity burden and prognosis, ASA and EF may be more useful in managing perioperative care [25]. However, several studies have found that the ASA score is closely linked to death rates and can be utilized as a marker for chronic diseases [20,26,27]. In a comorbidity study by Xing et al. [8], the CCI was identified as a meaningful predictive factor for predicting mortality rates. In our study, the ASA score, EF, and CCI were significantly associated with mortality. However, these parameters were not identified as independent risk factors in the multivariate risk model within the first 6 months. The presence of pre-existing chronic conditions may increase postoperative mortality by predisposing patients to complications such as pneumonia, acute renal failure, myocardial infarction, or pulmonary embolism [28,29]. Therefore, identifying and managing chronic conditions before surgery, along with implementing preventive measures, may help reduce postoperative mortality rates.

The increasing number of comorbidities, combined with the inadequacy of physiological compensation systems, may increase the requirement for postoperative ICU stays. Giummarra et al. [30] showed a substantial mortality risk in intensive care patients. In this study, a significant association was found between increased intensive care hospitalization and length of stay, although it was not identified to be a risk factor.

While several studies have focused on preoperative hypoalbuminemia [9,31], serum albumin levels may decrease following trauma, malnutrition, major surgery, or severe infection [32]. As an indicator or of inflammatory status, albumin levels may reflect clinical worsening or improvement depending on whether appropriate treatment is provided [32]. Untreated hypoalbuminemia has been associated with liver cirrhosis, nephrotic syndrome, sepsis, and increased mortality [33]. However, because the effectiveness of albumin replacement therapy remains inconclusive, management should primarily target the underlying cause [34]. Replacement therapy is generally recommended when albumin levels fall below 2 g/dL in patients with sepsis or major chronic illnesses [35]. In our study, patients with severe hypoalbuminemia and significant chronic comorbidities received albumin replacement therapy, whereas those with mild hypoalbuminemia were managed with nutritional support. Nonetheless, achieving target albumin levels through replacement therapy proved challenging, and some patients again demonstrated low albumin levels during post-discharge follow-up. Importantly, not only albumin replacement therapy but also management of the underlying conditions contributing to hypoalbuminemia may help reduce mortality.

A reduction in albumin levels has been shown to impair both functional status and mortality [9,36,37]. Sim et al. [31] demonstrated that when the albumin cutoff value is <3.5 g/dL, function and quality of life are lower before hip fracture due to muscle dysfunction, and recovery is delayed when measured with HHS after surgery. In this study, albumin levels and HHS scores declined in both groups following surgery, with Group 2 retaining higher levels.

Low albumin levels can lead to mortality through a variety of systemic consequences. Lee et al. [37] found that age, male gender, chronic respiratory disorders, delayed surgery, and low blood albumin levels are significant risk factors. Wiedermann’s study found that albumin insufficiency, which plays a crucial role in antimicrobial defense and repair, can induce wound healing issues, wound infection, sepsis, and septic shock alone [38]. Kishawi et al. [39] found a significant difference in the development of problems between patients with normal preoperative albumin levels and those with low albumin levels in the first 30 days post-surgery. In our study, preoperative albumin values were found to significantly influence mortality, although they were not identified as independent risk factors. Another study found that a 0.1 g/dL decline, independent of cutoff values, was related to a significant increase in problems, infections, and rehospitalizations [40]. We also observed that fluctuations in albumin levels during the first 6 months posed a mortality risk. Specifically, each 1 g/dL increase in albumin levels was associated with a significant reduction in mortality rates.

However, this study has limitations. First, this work is a retrospective and single-institution study. Second, patient numbers are limited. Third, the BMI of patients was not documented or assessed. Fourth, although the surgical procedure was standardized, it was performed by 20 different surgeons, which may have introduced variability. In addition, important preoperative parameters such as nutritional status and muscle mass were not adequately assessed. Finally, a lack of data regarding the post-discharge general health of deceased patients—particularly their nutritional intake and mobility—represents a major limitation of this study. Large-scale prospective studies should be performed to confirm these early findings.

## 5. Conclusions

In conclusion, this study demonstrates a statistically significant inverse association between postoperative serum albumin levels and early mortality within the first 6 months following hip fracture surgery. Given the biological plausibility of this association and the consistency of our findings with previous literature, serum albumin may serve as a valuable prognostic biomarker for postoperative risk stratification, particularly among elderly patients with high comorbidity burdens. Although causality cannot be inferred due to the retrospective study design, our results highlight the clinical importance of routine perioperative monitoring of albumin levels. Incorporating early nutritional assessment and targeted nutritional interventions into perioperative care protocols may improve survival outcomes in this vulnerable patient population.

## Figures and Tables

**Table 1:** Distribution of patients according to basic demographic, clinical, and perioperative characteristics.

Characteristic	Group 1 (*n* = 160)	Group 2 (*n* = 67)	*p* Value
Age, yr, med (min–max)	81 (64–97)	86 (65–96)	0.001 ^a^
Gender, *n* (%)			0.875 ^b^
Male	51 (31.9)	20 (29.9)	
Female	109 (68.1)	47 (70.1)	
Side, *n* (%)			0.772 ^b^
Right	85 (53.1)	37 (55.2)	
Left	75 (46.9)	30 (44.8)	
Fracture site, *n* (%)			0.307 ^b^
Femoral neck	91 (56.9)	43 (64.2)	
Intertrochanteric fracture	69 (43.1)	24 (35.8)	
Hospitalization duration, days, median (min–max)	6 (2–30)	6 (2–27)	0.617 ^a^
Intensive care status, *n* (%)			0.009 ^b^
No	88 (55)	24 (35.8)	
Yes	72 (45)	43 (64.2)	
Intensive care duration, days, median (min–max)	1 (0–15)	2 (0–25)	0.006 ^a^
Follow-up duration, months, median (min–max)	21 (1–33)	2 (1–5)	0.001 ^a^
Time from trauma to surgery, days, median (min–max)	1 (0–27)	1 (1–7)	0.278 ^a^
Operation duration, min, median (min–max)	75 (30–150)	70 (40–145)	0.233 ^a^
ASA classification, *n* (%)			0.002 ^b^
ASA 2	29 (18.1)	4 (6)	
ASA 3	113 (70.6)	45 (67.2)	
ASA 4	18 (11.3)	18 (26.9)	
Charlson Comorbidity Index, median (min–max)	5 (1–12)	6 (3–10)	0.002 ^a^
Hypertension, *n* (%)	70 (44)	48 (71.6)	0.001 ^b^
COPD, *n* (%)	20 (12.5)	28 (41.8)	0.001 ^b^
Chronic heart disease, *n* (%)	22 (14)	39 (58.2)	0.001 ^b^
EF, median (min–max)	60 (30–65)	55 (30–65)	0.032 ^a^
Chronic renal disease, *n* (%)	9 (5.6)	16 (23.9)	0.001 ^b^
Ischemic stroke, *n* (%)	19 (11.8)	20 (29.8)	0.001 ^b^
Diabetes mellitus, *n* (%)	26 (16.3)	29 (43.3)	0.001 ^b^
Cancer, *n* (%)	4 (2.5)	9 (13.4)	0.003 ^c^
Dementia, *n* (%)	12 (7.5)	23 (34.3)	0.001 ^b^
Osteoporosis, *n* (%)	107 (66.9)	55 (82.1)	0.021 ^a^
HHS (mean ± *SD*)	80 (70–89)	68 (45–74)	0.001 ^a^
Complications, *n* (%)			
Dislocation	4 (2.5)	2 (3)	0.385 ^c^
Infection	9 (5.65)	6 (8.9)	

Note. ASA, American Society of Anesthesiologists; COPD, chronic obstructive pulmonary disease; HHS, Harris Hip Score; *p* < 0.05; min, minimum; max, maximum. ^a^ Mann–Whitney *U* test. ^b^ Pearson Chi-square test. ^c^ Fisher’s Exact test.

**Table 2:** Comparative Analysis of Preoperative and Postoperative Laboratory Parameters Between Groups.

Parameters	Group 1 (*n* = 160)	Group 2 (*n* = 67)	*p*
Preoperative NLR median (min–max)	7.24 (0.03–33.3)	8.67 (1.86–29.2)	0.472 ^a^
Postoperative NLR median (min–max)	8.88 (1.88–40)	8.7 (1.71–33.4)	0.680 ^a^
Preoperative albumin median (min–max)	38 (25–48)	36 (21–48)	0.001 ^a^
Postoperative albumin median (min–max)	31 (21–40)	29 (17–37)	0.001 ^a^
Preoperative hemoglobin median (min–max)	12.45 (9–17)	12.5 (10–16)	0.477 ^a^
Postoperative hemoglobin median (min–max)	10.2 (7–14)	10.1 (7–13)	0.418 ^a^
Albumin < 29.5	44 (27.5)	39 (58.2)	0.001 ^b^
Albumin > 29.6	116 (72.5)	28 (41.8)	

Note. EF, ejection fraction; max, maximum; min, minimum; NLR, neutrophil–lymphocyte ratio. ^a^ Mann–Whitney *U* test. ^b^ Pearson Chi-square test.

**Table 3:** Evaluation of parameters affecting mortality in the first 6 months using univariate binary logistic regression analysis.

Variables	β	SE	*p* Value	OR	95% CI
Gender	−0.095	0.316	0.764	0.909	0.489–1.691
Time from trauma to surgery	−0.114	0.09	0.204	0.892	0.747–1.064
Preoperative NLR	0.009	0.023	0.689	1.009	0.965–1.05
Postoperative NLR	−0.015	0.023	0.512	0.985	0.941–1.031
Fracture site	−0.306	0.301	0.308	0.736	0.408–1.327
Operation duration	−0.008	0.007	0.244	0.992	0.980–1.005
Preoperative hemoglobin	−0.078	0.094	0.405	0.925	0.770–1.111
Postoperative hemoglobin	−0.102	0.103	0.321	0.903	0.737–1.105
Hospitalization duration	0.017	0.031	0.581	1.017	0.958–1.080
Age	0.061	0.02	0.003	1.063	1.022–1.106
EF	−0.059	0.024	0.013	0.943	0.900–0.988
ASA 3	1.06	0.562	0.059	2.887	0.960–8.682
ASA 4	1.981	0.629	0.002	7.25	2.113–24.872
Preoperative albumin	−0.156	0.039	0.001	0.856	0.793–0.924
Postoperative albumin	−0.208	0.045	0.001	0.812	0.744–0.887
CCI	0.247	0.092	0.007	1.28	1.068–1.535
Intensive care status	0.784	0.3	0.009	2.19	1.216–3.945
Intensive care duration	0.124	0.047	0.008	1.132	1.033–1.240

Note. NLR, neutrophil lymphocyte ratio; ASA, American Society of Anesthesiologists; CCI, Charlson Comorbidity Index; EF, ejection fraction; OR, odds ratio; SE, standard error; 95% CI, 95% confidence interval; β, Standardized Regression Coefficient.

**Table 4:** Multivariate binary logistic regression analysis of parameters affecting mortality at 1, 3 and 6 months.

Variables	β	SE	*p* Value	OR	95% CI
1st month					
Constant	6.789	3.556	0.056	887.859	
Postoperative albumin	−0.190	0.075	0.011	0.827	0.714–0.958
3rd month					
Constant	4.235	3.07	0.167	69.05	
Postoperative albumin	−0.207	0.066	0.002	0.813	0.715–0.925
6th month					
Constant	3.469	2.911	0.233	32.108	
Postoperative albumin	−0.180	0.061	0.003	0.835	0.741–0.941

Note. 95% CI, 95% confidence interval; β, Standardized Regression Coefficient; OR, odds ratio; SE, standard error.

**Table 5:** Determination of Cut-off Values for Mortality-Associated Factors Using ROC Curve Analysis.

Risk Factor	AUC (95% CI)	*p* Value	Cutoff	Sensitivity (%)	Specificity (%)	J
Preoperative albumin	0.688 (0.613–0.764)	0.001	3.75	70.1	64.4	0.345
Postoperative albumin	0.700 (0.626–0.774)	0.001	2.95	72.5	58.2	0.307
Age	0.634 (0.555–0.714)	0.001	81.5	70.1	53.1	0.233
EF	0.585 (0.503–0.666)	0.044	52.5	23.9	87.5	0.114
ASA	0.618 (0.538–0.697)	0.005	3.5	26.9	88.7	0.156
CCI	0.629 (0.554–0.704)	0.002	5.5	56.7	63.1	0.198
Intensive care duration	0.608 (0.527–0.689)	0.01	1.5	59.7	61.9	0.216

Note. ASA, American Society of Anesthesiologists; CCI, Charlson Comorbidity Index; EF, ejection fraction; ROC, Receiver Operating Characteristic; 95% CI, 95% confidence interval; AUC, areas under the curve; J, Youden Index.

## Data Availability

The raw data supporting the conclusions of this article will be made available by the corresponding author upon request.
